# Supplementary material for: Insomnia symptoms and neurofunctional correlates among adults receiving buprenorphine for opioid use disorder
Source: PLoS One. 2024 Jun 13;19(6):e0304461. doi: 10.1371/journal.pone.0304461 (PMC11175529; doi:10.1371/journal.pone.0304461)
Supplement: S1 Appendix — Contains all supporting information (S1–S5 Tables). (DOCX) [file pone.0304461.s001.docx]

**Supporting information**

**S1 Table. 5-Trial Adjusted Delay Discounting Task Details and Alternative Outcomes.**

**S2 Table. Differences in Neurocognitive Performance and OUD Treatment Perspectives as a Function of Insomnia Symptom Status, Imputed Results.**

**S3 Table. Adjusted Differences in Neurocognitive Performance as a Function of Insomnia Symptom Status (Select Results).**

**S4 Table. Adjusted Differences in Neurocognitive Performance as a Function of Insomnia Symptom Status with Higher Insomnia Severity Index Cutoff Score (Select Results).**

**S5 Table. Differences in Neurocognitive Performance and OUD Treatment Perspectives as a Function of Insomnia Symptom Status (Continuous Variable Specification)**

**S1. 5-Trial Adjusted Delay Discounting Task Details and Alternative Outcomes**

Due to a coding issue with the software (REDCAP) used to implement the survey, instead of the 5-trial adjusting delay discounting task being presented in an “adaptive” manner, choices were presented in sequential order. This meant that instead of answering only 5 questions, with the next question a participant viewed being determined by their response to the previous question, each participant answered all 31 possible questions in the task (see below for questions in the order they were answered):

*In this game, you will be asked to make choices between getting $5 right NOW, or getting $10 some time in the future.*

*Select the option that you would prefer.*

*This game takes about 1 minute to play. There are no right or wrong answers.*

1. *Would you rather have…?*

*$5 now*

*$10 in 3 weeks*

1. *Would you rather have…?*

*$5 now*

*$10 in 1 day*

1. *Would you rather have…?*

*$5 now*

*$10 in 2 years*

1. *Would you rather have…?*

*$5 now*

*$10 in 4 hours*

1. *Would you rather have…?*

*$5 now*

*$10 in 4 days*

1. *Would you rather have…?*

*$5 now*

*$10 in 4 months*

1. *Would you rather have…?*

*$5 now*

*$10 in 8 years*

1. *Would you rather have…?*

*$5 now*

*$10 in 2 hours*

1. *Would you rather have…?*

*$5 now*

*$10 in 9 hours*

1. *Would you rather have…?*

*$5 now*

*$10 in 2 months*

1. *Would you rather have…?*

*$5 now*

*$10 in 8 months*

1. *Would you rather have…?*

*$5 now*

*$10 in 2 days*

1. *Would you rather have…?*

*$5 now*

*$10 in 1.5 weeks*

1. *Would you rather have…?*

*$5 now*

*$10 in 4 years*

1. *Would you rather have…?*

*$5 now*

*$10 in 18 years*

1. *Would you rather have…?*

*$5 now*

*$10 in 1 hour*

1. *Would you rather have…?*

*$5 now*

*$10 in 3 hours*

1. *Would you rather have…?*

*$5 now*

*$10 in 6 hours*

1. *Would you rather have…?*

*$5 now*

*$10 in 12 hours*

1. *Would you rather have…?*

*$5 now*

*$10 in 1 month*

1. *Would you rather have…?*

*$5 now*

*$10 in 3 months*

1. *Would you rather have…?*

*$5 now*

*$10 in 6 months*

1. *Would you rather have…?*

*$5 now*

*$10 in 1 year*

1. *Would you rather have…?*

*$5 now*

*$10 in 1.5 days*

1. *Would you rather have…?*

*$5 now*

*$10 in 3 days*

1. *Would you rather have…?*

*$5 now*

*$10 in 1 week*

1. *Would you rather have…?*

*$5 now*

*$10 in 2 weeks*

1. *Would you rather have…?*

*$5 now*

*$10 in 3 years*

1. *Would you rather have…?*

*$5 now*

*$10 in 5 years*

1. *Would you rather have…?*

*$5 now*

*$10 in 12 years*

1. *Would you rather have…?*

*$5 now*

*$10 in 25 years*

To account for this issue, we opted to analyze participants responses by only considering their responses to the items they *should* have seen on the task according to the figure below. Final *k* values (presented in taken from Koffarnus and Bickel, 2014) were assigned based on the precise branch they followed. For instance, if someone picked only delayed rewards (followed the blue lines down [Q16🡪Q8🡪Q4🡪Q2🡪Q1🡪*k*]), their k-value would be equal to 17.


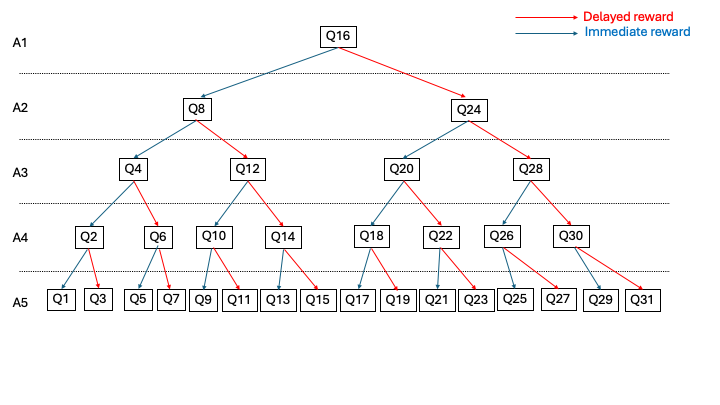


We also made the determination to present results from the 5-DD task in terms of ED50, which is equal to 1/*k*. The reason for this decision was that *k* is a unitless expression that is difficult to interpret except in ordinal terms and no transformations of *k* (e.g., ln[*k*]) yielded a normal distribution. In contrast, ED50 has an easily understood interpretation (i.e., the amount of time required for a reinforcer to lose 50% of its subjective value). Below, we present results from the 5-DD in terms of ED50, *k,* and ln(*k*).

|  | **Minimal Evidence of Insomnia Symptoms**  **(INS-; ISI:** $\boldsymbol{\leq}$**10)** | **Evidence of Insomnia Symptoms**  **(INS+; ISI:** $\boldsymbol{\geq}$**11)** | **u-test**  **p-value** | **t-test**  **p-value** |
| --- | --- | --- | --- | --- |
| **ED50** | 1715.78 (2628.3) | 1899.55 (2415.2) | 0.2138 | 0.6928 |
| ***k*** | 0.52 (3.4) | 0.46 (2.93) | 0.2138 | 0.9074 |
| **ln(*k*)** | -5.80 (2.6) | -6.26 (2.5) | 0.2138 | 0.3280 |

NOTE: A total of 120 participants were included in these analyses. The above presents mean (standard deviation) as well as the corresponding p-value from a u-test (non-parametric) and t-test (parametric) comparing the two insomnia symptom status groups.

**S2. Differences in Neurocognitive Performance and OUD Treatment Perspectives as a Function of Insomnia Symptom Status, Imputed Results**

| **Measure** | **Insomnia Severity Index Score**  **Beta Coefficient [Std Error]** | **p-value** |
| --- | --- | --- |
| **Short UPPS-P Impulsive Behavior Scale** |  |  |
| *Negative urgency* | 1.15 [0.7] | 0.097 |
| *Lack of perseverance* | 0.48 [0.5] | 0.314 |
| *Lack of premeditation* | 0.40 [0.5] | 0.424 |
| *Sensation seeking* | 0.48 [0.7] | 0.482 |
| *Positive urgency* | 0.55 [0.7] | 0.409 |
| **5-Trial Adjusting Delay Discounting Task (Effective Delay 50)** | 223.31 (499.1) | 0.656 |
| **Multidimensional Assessment of Interoceptive Awareness** |  |  |
| *Noticing* | 0.75 [0.3] | 0.005 |
| *Not distracting* | -0.33 [0.2] | 0.128 |
| *Not worrying* | -0.16 [0.2] | 0.403 |
| *Attention regulation* | -0.08 [0.2] | 0.755 |
| *Emotional awareness* | 0.45 [0.2] | 0.066 |
| *Self-regulation* | 0.07 [0.3] | 0.779 |
| *Body listening* | 0.27 [0.2] | 0.253 |
| *Trusting* | -0.32 [0.3] | 0.265 |
| **Patient Health Questionnaire-9** | 6.17 [1.2] | <0.001 |
| **General Anxiety Disorder-7** | 4.84 [1.2] | <0.001 |
| **Post-Traumatic Stress Disorder Checklist** | 16.54 [4.1] | <0.001 |
| **PROMIS-10 Health Scale** |  |  |
| *Global Physical Health* | -0.57 [0.4] | 0.114 |
| *Global Mental Health* | 1.04 [0.5] | 0.027 |
| **Distress Tolerance Scale** |  |  |
| *Tolerance* | 0.52 [0.2] | 0.023 |
| *Absorbance* | 0.58 [0.2] | 0.013 |
| *Appraise* | 0.39 [0.2] | 0.031 |
| *Regulation* | 0.16 [0.2] | 0.457 |
| **Buss-Perry Aggression Scale** | -5.12 [4.1] | 0.217 |
| *Physical aggression* | -1.56 [1.5] | 0.302 |
| *Verbal aggression* | -0.36 [0.9] | 0.687 |
| *Anger* | -1.12 [1.1] | 0.315 |
| *Hostility* | 0.73 [1.8] | 0.687 |
| **Snaith-Hamilton Pleasure Scale** | 0.24 [0.5] | 0.611 |
| **Metacognition Questionnaire-30** | 6.27 [3.2] | 0.054 |
| *Lack of confidence* | 1.42 [0.8] | 0.099 |
| *Positive worry* | 0.76 [0.8] | 0.347 |
| *Cognitive confidence* | 1.02 [0.9] | 0.263 |
| *Uncontrollability* | 2.20 [0.9] | 0.016 |
| *Controlling thoughts* | 0.53 [0.8] | 0.532 |
| **Poor sleep interferes with OUD treatment** | 0.88 [0.2] | <0.001 |
| **Improved sleep would assist with OUD treatment** | 1.17 [0.2] | <0.001 |

NOTE: Results reported here were based on 10 imputed datasets (N=129) created using multivariate normal distribution (MVN) regression; predictor variables were those demographic characteristics with complete data (i.e., insomnia symptom severity index category, gender, employment status, educational attainment, and buprenorphine dose). ISI groups were compared using simple linear regression, which effectively reduces to a t-test, with the resulting beta coefficient estimate [Standard Error] reported (i.e., difference in average score between groups). The reference category in all regressions was the “minimal evidence of clinically significant insomnia” group. The Distress Tolerance Scale was reverse coded, such that higher scores indicate more problematic stress reactions within each subdomain. Bolded lines indicate total or summary scores for scales utilized in the PhAB-Brief; italicized lines indicate scores from subscales.

**S3. Adjusted Differences in Neurocognitive Performance as a Function of Insomnia Symptom Status (Select Results)**

|  | **MAIA** *Noticing Subscale* | **PHQ9**  *Total Score* | **GAD7**  *Total Score* | **PCL5**  *Total Score* | **PROMIS10** *Global Mental Health* | **DTS** *Tolerance Subscale* | **DTS**  *Appraisal Subscale* | **DTS**  *Absorbance Subscale* | **MCQ30**  *Uncontrollability and Danger*  *Subscale* |
| --- | --- | --- | --- | --- | --- | --- | --- | --- | --- |
| **Insomnia Symptom Status Category (REFERENCE GROUP: Minimal Evidence of Insomnia Symptoms (INS-; ISI:** $\boldsymbol{\leq}$**10))** | | | | | | | | | |
| **Evidence of Insomnia Symptoms (INS+; ISI:**$\boldsymbol{\geq}$**11)** | 0.52*  (0.3) | 6.05****  (1.3) | 4.86****  (1.2) | 17.17****  (4.3) | 1.38***  (0.5) | 0.46**  (0.2) | 0.41**  (0.2) | 0.56**  (0.2) | 2.25**  (1.0) |

NOTE: ********p<0.001, ***p<0.01, **p<0.05, *p<0.10. Individual regression results reported above were derived from Linear Regressions (separate regression for each outcome), modeled with robust standard errors. A total of 129 participants were included in these analyses (i.e., the imputed dataset). Results above present the beta coefficient and standard error for each variable, effectively the difference in the average score for each outcome between groups. The reference category in all regressions was the “minimal evidence of clinically significant insomnia” group. Because imputed datasets were used for these analysis, we were unable to perform seemingly unrelated regressions to account for correlation in the error term across equations. Though not shown above, each regression also controlled for gender, age, race, education, and buprenorphine dosage (full regression results are available upon request).

MAIA = Multidimensional Assessment of Interoceptive Awareness

PHQ9 = Patient Health Questionnaire-9

GAD7 = Generalized Anxiety Disorder-7

PCL5 = Post-Traumatic Stress Check List-5

PROMIS10 = Patient Reported Outcome Measurement Information System

DTS = Distress Tolerance Scale

MCQ30 = Metacognition Questionnaire 30

**S4. Adjusted Differences in Neurocognitive Performance as a Function of Insomnia Symptom Status with Higher Insomnia Severity Index Cutoff Score (Select Results)**

|  | **MAIA** *Noticing Subscale* | **PHQ9**  *Total Score* | **GAD7**  *Total Score* | **PCL5**  *Total Score* | **PROMIS10** *Global Mental Health* | **DTS** *Tolerance Subscale* | **DTS**  *Appraisal Subscale* | **DTS**  *Absorbance Subscale* | **MCQ30**  *Uncontrollability and Danger*  *Subscale* |
| --- | --- | --- | --- | --- | --- | --- | --- | --- | --- |
| **Insomnia Symptom Status Category (REFERENCE GROUP: Minimal Evidence of Insomnia Symptoms (INS-; ISI:** $\boldsymbol{\leq}$**10))** | | | | | | | | | |
| **Evidence of Insomnia Symptoms (INS+; ISI:**$\boldsymbol{\geq}$**11)** | 0.61**  (0.3) | 6.42****  (1.2) | 5.83****  (1.1) | 15.69****  (4.0) | 1.64****  (0.5) | 0.46**  (0.2) | 0.42**  (0.2) | 0.53**  (0.2) | 1.57  (1.0) |

NOTE**: ******p<0.001, ***p<0.01, **p<0.05, *p<0.10. The cut off scores used here for the insomnia severity index were: minimal-to-subthreshold insomnia symptoms (raw scores: 0-14; N=70 [54.2%]) and moderate-to-severe insomnia symptoms (raw score: 15-28; N=59 [45.7%]). Individual regression results reported above were derived from Linear Regressions (separate regression for each outcome), modeled with robust standard errors. A total of 129 participants were included in these analyses (i.e., the imputed dataset). Results above present the beta coefficient and standard error for each variable, effectively the difference in the average score for each outcome between groups. The reference category in all regressions was the “minimal evidence of clinically significant insomnia” group. Because imputed datasets were used for these analysis, we were unable to perform seemingly unrelated regressions to account for correlation in the error term across equations. Though not shown above, each regression also controlled for gender, age, race, education, and buprenorphine dosage (full regression results are available upon request).

MAIA = Multidimensional Assessment of Interoceptive Awareness

PHQ9 = Patient Health Questionnaire-9

GAD7 = Generalized Anxiety Disorder-7

PCL5 = Post-Traumatic Stress Check List-5

PROMIS10 = Patient Reported Outcome Measurement Information System

DTS = Distress Tolerance Scale

MCQ30 = Metacognition Questionnaire 30

**S5. Differences in Neurocognitive Performance and OUD Treatment Perspectives as a Function of Insomnia Symptom Status (Continuous Variable Specification)**

| **Measure** | **Insomnia Severity Index Score**  **Beta Coefficient [Std Error]** | **p-value** |
| --- | --- | --- |
| **Short UPPS-P Impulsive Behavior Scale** |  |  |
| *Negative urgency* | 0.056 (0.05) | 0.221 |
| *Lack of perseverance* | 0.052 (0.03) | 0.127 |
| *Lack of premeditation* | 0.032 (0.04) | 0.400 |
| *Sensation seeking* | 0.015 (0.04) | 0.708 |
| *Positive urgency* | 0.032 (0.04) | 0.462 |
| **5-Trial Adjusting Delay Discounting Task (Effective Delay 50)** | -25.085 (34.9) | 0.474 |
| **Multidimensional Assessment of Interoceptive Awareness** |  |  |
| *Noticing* | 0.041 (0.02) | 0.034 |
| *Not distracting* | -0.028 (0.01) | 0.062 |
| *Not worrying* | -0.016 (0.01) | 0.137 |
| *Attention regulation* | -0.002 (0.02) | 0.908 |
| *Emotional awareness* | 0.022 (0.02) | 0.221 |
| *Self-regulation* | -0.005 (0.02) | 0.794 |
| *Body listening* | 0.023 (0.02) | 0.181 |
| *Trusting* | -0.030 (0.02) | 0.173 |
| **Patient Health Questionnaire-9** | 0.540 (0.06) | <0.001 |
| **General Anxiety Disorder-7** | 0.432 (0.06) | <0.001 |
| **Post-Traumatic Stress Disorder Checklist** | 1.195 (0.22) | <0.001 |
| **PROMIS-10 Health Scale** |  |  |
| *Global Physical Health* | -0.015 (0.02) | 0.504 |
| *Global Mental Health* | 0.158 (0.03) | <0.001 |
| **Distress Tolerance Scale** |  |  |
| *Tolerance* | 0.039 (0.01) | 0.006 |
| *Absorbance* | 0.042 (0.01) | 0.005 |
| *Appraise* | 0.030 (0.01) | 0.011 |
| *Regulation* | 0.015 (0.01) | 0.309 |
| **Buss-Perry Aggression Scale** | 0.028 (0.28) | 0.922 |
| *Physical aggression* | 0.007 (0.10) | 0.938 |
| *Verbal aggression* | 0.016 (0.06) | 0.780 |
| *Anger* | -0.020 (0.08) | 0.796 |
| *Hostility* | 0.119 (0.12) | 0.329 |
| **Snaith-Hamilton Pleasure Scale** | 0.019 (0.03) | 0.594 |
| **Metacognition Questionnaire-30** | 0.435 (0.21) | 0.043 |
| *Lack of confidence* | 0.106 (0.05) | 0.045 |
| *Positive worry* | 0.027 (0.05) | 0.620 |
| *Cognitive confidence* | 0.034 (0.06) | 0.569 |
| *Uncontrollability* | 0.189 (0.06) | 0.001 |
| *Controlling thoughts* | 0.095 (0.06) | 0.105 |
| **Poor sleep interferes with OUD treatment** | 0.077 (0.02) | <0.001 |
| **Improved sleep would assist with OUD treatment** | 0.086 (0.02) | <0.001 |

NOTE: A total of 129 participants were included in these analyses, though the sample size for each outcome varies due to participants not answering every question for a given instrument (i.e., list-wise deletion). Sample sizes are equivalent to those reported in Table 2 for each outcome. Results above present the beta coefficient estimate [Standard Error] for the ISI total score as a predictor for each neurofunctional outcome controlling for demographic characteristics (gender, age, race, educational attainment) and buprenorphine dose (covariate estimates not shown). As opposed to other analyses presented in Table 2 and supplementary tables S1-S4, here the ISI score was entered into the model as a continuous rather than a categorical variable. The Distress Tolerance Scale was reverse coded, such that higher scores indicate more problematic stress reactions within each subdomain. Bolded lines indicate total or summary scores for scales utilized in the PhAB-Brief; italicized lines indicate scores from subscales.
