# Supplementary material for: Insomnia symptoms and neurofunctional correlates among adults receiving buprenorphine for opioid use disorder
Source: PLoS One. 2024 Jun 13;19(6):e0304461. doi: 10.1371/journal.pone.0304461 (PMC11175529; doi:10.1371/journal.pone.0304461)
Supplement: Attachment — Submitted filename: PONE-D-23-39561 Review.docx [file pone.0304461.s002.docx]

Review notes

**COMMENTS TO THE AUTHORS:**

The authors of this manuscript aimed to examine the relationship between insomnia symptoms and several areas of neurocognitive performance and mental health symptoms in persons with opioid use disorder (OUD) who are receiving buprenorphine. The authors review the growing body of literature on various aspects of sleep disruption, including insomnia, in those who are receiving medications for opioid use disorder and provide rationale for the need to examine how this disruption relates to neurofunctional correlates. A continuous scale of insomnia was dichotomized by a cutoff score to differentiate those with and without clinically significant insomnia symptoms. This could be seen as a limiting factor, though the authors provided rationale and pointed to precedent in other literature. In 129 persons receiving buprenorphine they found the majority (58.9%) were experiencing clinical significant insomnia symptoms, and higher symptoms were related to symptoms of depression, anxiety, post-traumatic stress, stress intolerance, unhelpful metacognition, and interoceptive awareness. Further, those with significant insomnia symptoms endorsed sleep as interfering with OUD treatment and that improving sleep would assist with OUD treatment.

Strengths of the manuscript include the use of validated measures, a fairly large sample size, and addressing the understudied area of the relationship between sleep and neurofunctioning in OUD recovery. Moreover, the authors identified a specific sleep problem (insomnia) rather than generically discussing “sleep disturbance” or “poor sleep.” This nuance is very important in the literature, as the more broad terms do little to advance the field. Clarifying which sleep disorder symptoms are being assessed helps focus to be narrowed to specific, targetable modifiable risk factors that can be addressed in future studies. This manuscript could be improved if the authors considered and/or edited as follows:

- If word count allows, this paper would benefit from a little more explanation in the Introduction. Specifically P. 3, paragraph 2, lines 55-58 references that neurofunctional performance can be impacted and minimal research has been done in this area, but does not break down the various aspects of functioning/performance that will be looked at and why. This is the gap in the literature this study is proposing to fill and should therefore probably be expanded a little more.
- On page 5 the reporting of ISI “INS+” and “INS-“ randomly switches to serif font rather than sans serif
- It has been proposed by some that sleep should stabilize over time after dose is stabilized, thus how long they have been receiving buprenorphine would impact sleep, and in theory these other variables would improve. If the authors have data on how long participants have been engaged with MOUD it may be helpful to present the mean amount of time or range of time. That would allow demonstration that insomnia can persist for years if not directly addressed.
- Similar to the Introduction, the Discussion would benefit from elaboration. The non-significant findings are just as interesting as the significant ones. There is a good bit of literature about how insomnia often correlates with subjective measures of cognitive performance and mental health symptoms, but the relationship with objective measures is less clear. That seems to have been mirrored in this group as well and hypotheses around that and its implications for treatment would enrich the Discussion section.
- Running so many t-tests without accounting for family-wise error is a limitation that should be listed.
